# Cytokine Storm in COVID-19: Exploring IL-6 Signaling and Cytokine-Microbiome Interactions as Emerging Therapeutic Approaches

**DOI:** 10.3390/ijms252111411

**Published:** 2024-10-24

**Authors:** Tudorita Gabriela Paranga, Ivona Mitu, Mariana Pavel-Tanasa, Manuel Florin Rosu, Ionela-Larisa Miftode, Daniela Constantinescu, Maria Obreja, Claudia Elena Plesca, Egidia Miftode

**Affiliations:** 1 Department of Infectious Diseases (Internal Medicine II), Faculty of Medicine, Grigore T. Popa University of Medicine and Pharmacy, 700115 Iasi, Romania; tudorita.paranga@umfiasi.ro (T.G.P.); ionela-larisa.miftode@umfiasi.ro (I.-L.M.); maria-obreja@umfiasi.ro (M.O.); claudia-elena-g-plesca@umfiasi.ro (C.E.P.); egidia.miftode@umfiasi.ro (E.M.); 2 St. Parascheva Clinical Hospital for Infectious Diseases, 700116 Iasi, Romania; 3 Department of Morpho-Functional Sciences II, Grigore T. Popa University of Medicine and Pharmacy, 700115 Iasi, Romania; ivona.mitu@umfiasi.ro; 4 Department of Immunology, Faculty of Medicine, Grigore T. Popa University of Medicine and Pharmacy, 700115 Iasi, Romania; d.constantinescu@umfiasi.ro; 5 Laboratory of Immunology, St. Spiridon County Clinical Emergency Hospital, 700101 Iasi, Romania; 6 Department of Preventive Medicine and Interdisciplinarity, Faculty of Medicine, University of Medicine and Pharmacy Grigore. T. Popa, 700115 Iasi, Romania

**Keywords:** IL-6, TNF-α, IFN-γ, cytokine storm, COVID-19, tocilizumab

## Abstract

IL-6 remains a key molecule of the cytokine storms characterizing COVID-19, exerting both proinflammatory and anti-inflammatory effects. Emerging research underscores the significance of IL-6 trans-signaling over classical signaling pathways, which has shifted the focus of therapeutic strategies. Additionally, the synergistic action of TNF-α and IFN-γ has been found to induce inflammatory cell death through PANoptosis, further amplifying the severity of cytokine storms. Long COVID-19 patients, as well as those with cytokine storms triggered by other conditions, exhibit distinct laboratory profiles, indicating the need for targeted approaches to diagnosis and management. Growing evidence also highlights the gut microbiota’s crucial role in modulating the immune response during COVID-19 by affecting cytokine production, adding further complexity to the disease’s immunological landscape. Targeted intervention strategies should focus on specific cytokine cutoffs, though accurate cytokine quantification remains a clinical challenge. Current treatment strategies are increasingly focused on inhibiting IL-6 trans-signaling, which offers promise for more precise therapeutic approaches to manage hyperinflammatory responses in COVID-19. In light of recent discoveries, this review summarizes key research findings on cytokine storms, particularly their role in COVID-19 and other inflammatory conditions. It explores emerging therapeutic strategies targeting cytokines like IL-6, TNF-α, and IFN-γ, while also addressing open questions, such as the need for better biomarkers to detect and manage cytokine storms. Additionally, the review highlights ongoing challenges in developing targeted treatments that mitigate hyperinflammation without compromising immune function, emphasizing the importance of continued research in this field.

## 1. Introduction

This year marks four years since the COVID pandemic began, and although much has been documented, many unanswered questions remain about the intricate interplay of molecular pathways that occur with the dysregulation of the immune system. Although the uncontrolled release of cytokines is clearly detrimental to the organism, leading to multiorgan failure and high mortality rates, a clear separation between a normal and a dysregulated immune reaction in the case of a severe infection still demands continuous research.

While cytokine storm (CS) characterizes the pathogeny of COVID-19, it is not specific to the SARS-CoV-2 infection, since this exaggerated immune reaction of the host organism has already been extensively linked to several diseases as a response to different pathogens, therapies, autoimmune disorders, and cancers [1]. In the 1990s, CS was first documented to be associated with graft-versus-host disease, and it was only later in the 2000s when the literature reported its critical role in infectious diseases caused by cytomegalovirus, influenza virus, Epstein–Barr virus-associated hemophagocytic lymphohistiocytosis, group A streptococcus, variola virus, or coronavirus. Interestingly, CS is also associated with non-infectious settings, notably called “macrophage activation system” in autoimmune diseases such as systemic lupus erythematosus and systemic juvenile idiopathic arthritis [2]. Moreover, CS is also reported as a response to immunotherapeutic agents such as specific T cell-activating antibodies [3,4], while it is also induced by immune checkpoint inhibitors (ICI). The latter targeted therapy response is known in oncology as cytokine release syndrome, which encompasses CS and highlights the pluripotent role of cytokines in human pathophysiology [4]. Therefore, cytokines still present an important focus in research, since understanding their roles could elucidate important cellular and molecular mechanisms to improve diagnosis, prognosis, and better understand disease progression.

Many factors contribute to the dysregulation of the host immune system in COVID-19, resulting in some patients being able to control the viral infection and develop asymptomatic, mild, or moderate forms, while others are unable to control the virus or limit the infection, leading to severe or critical forms, including ARDS with MODS or even death [1]. We have also previously shown that distinct profiles of cytokines and immune checkpoint molecules characterize disease severity and mortality in COVID-19 patients [5,6]. Thus, the scope of this review is to highlight recent advances in understanding CS in COVID-19 as an essential checkpoint guiding disease progression. We provide an in-depth analysis of the most relevant cytokines, the laboratory challenges in measuring them, and their intertwined roles, while exploring the progress in improving prognosis, disease staging, and the latest updates on treatment options.

## 2. The Hallmarks of Cytokine Storm in COVID-19

Cytokine storm is a life-threatening condition characterized by: clinical symptoms of systemic hyperinflammation, elevated levels of circulating cytokines, and multiple organ dysfunction caused by pathogens, various therapies, and monogenic and autoimmune disorders, even cancers (Table 1).

The underlying cause of CS influences the laboratory findings, since nonspecific markers of inflammation such as CRP, lactate dehydrogenase (LDH), ferritin, and blood counts are universally elevated and correlated with disease severity [1], whereas other serum cytokine levels seem to better correlate with specific pathologies. For instance, patients with sepsis-induced CS have higher levels of circulating IL-1β, procalcitonin and markers of endothelial damage than patients with CAR T-cell therapy-induced CS [1]. Alternatively, when the CS is caused by macrophage activation syndrome (MAF), the serum levels of interleukin 18 are elevated, while CS due to HLH is often characterized by elevated levels of TNF-a, interferon γ (IFN-γ), interleukin 1 (IL-1), IL-4, IL-6, IL-8, IL-10, IL-18, CXCL9, and CXCL10 [1]. In essence, investigating the underlying disease provides a specific and comprehensive biomarker panel that is key for clinicians in better evaluating CS.

COVID-19-associated CS shows elevated levels of IL-1β, IL-6, CXCL10, TNF-α, IFN-γ, and macrophage inflammatory protein (MIP) 1α and 1β, as well as monocytes chemoattractant protein 1 (MCP-1), granulocyte–macrophage colony-stimulating factor (GM-CSF), VEGF, and IL-10 [1,27] The levels of some of these cytokines were also found to correlate with disease severity [28]. Moreover, circulating activated CD4+ and CD8+ T cells and plasmablasts are observed, as well as usual abnormalities similar to other CS disorders, such as elevated CRP and D-dimer levels, renal dysfunction, hypoalbuminemia, and effusions [28]. Although Lucas et al. suggested that circulating levels of IL-6 and ferritin are less severely elevated in COVID-19 than in some of the other CS disorders [29], high IL-6 levels are nevertheless strongly associated with shorter survival in COVID-19 patient cohorts [1].

Conversely, lymphopenia is uncommon in CS disorders, but is a hallmark of severe COVID-19. Whether lymphocyte destruction or tissue infiltration is the underlying mechanism remains unclear [1]. It is well known that clotting issues can occur in CS disorders, but COVID-19-associated CS thromboembolic events are present to a higher rate [1]. Furthermore, Karki and Kanneganti observed a marked increase in TNF and IFN-γ levels in the sera of patients suffering from severe COVID-19 [2]. All these characteristics of CS in COVID-19 are the result of a relatively short period of research, but reflect a sustained global effort by researchers to better characterize the disease.

### 2.1. Key Proinflammatory Cytokines—Past and Present

In patients with COVID-19, cytokine storm is recognized as the primary factor contributing to disease severity and mortality [30]. Uncontrolled systemic inflammation, marked by the overproduction of inflammatory cytokines by immune cells, leads to diverse local biological consequences due to the disruption of negative feedback mechanisms within the immune system. Subsequently, these cytokines induce a feedforward loop on other immune cells, perpetuating their recruitment and leading to an exponential increase in inflammation and organ damage. In summary, this process entails a hyperactivation and continuous assault on the immune system. The principal cytokines involved in SARS-CoV-2 infection include interleukins, interferons, colony-stimulating factors, tumor necrosis factors, the chemokine family (CCL2, CCL3, CCL5, CXCL8, CXCL9, CXCL10, etc.), and growth factors. These cytokines are categorized based on their functions into proinflammatory (IL-1β, IL-6, IL-12, TNF-α, and IFN-γ) and anti-inflammatory modulators (IL-4, IL-10, IL-13, and TGF-β) [31]. Here, we focus on detailing the most relevant findings related to the cytokines targetable by specific therapies, as well as their interconnections with the microbiome.

#### 2.1.1. IL-6

IL-6 is a central cytokine in the inflammatory response to COVID-19, playing both proinflammatory and anti-inflammatory roles that significantly impact disease progression. IL-6 can mediate classic signaling through its attachment to membrane-bound IL-6R (mIL-6R) and trans-signaling with soluble IL-6R (sIL-6R), with low serum IL-6 levels favoring the anti-inflammatory classic pathway and elevated IL-6 levels shifting to the proinflammatory trans-signaling pathway, while activating a broader cell population [32]. IL-6 signaling promotes the recruitment and activation of various immune cells, including T cells, B cells, macrophages, and neutrophils, thereby amplifying the inflammatory response [32,33,34]. The dysregulation of this pathway is a prominent feature of the hyperinflammatory state caused by SARS-CoV-2 infection. Elevated IL-6 levels in COVID-19 patients are linked to the significant depletion of CD4+ T cells, CD8+ T cells, and natural killer cells, contributing to the compromised immune response [16,17]. Recent research highlights the importance of IL-6 trans-signaling, rather than the classic signaling, in driving the hyperinflammatory responses seen in severe COVID-19. This aberrant signaling pathway contributes to respiratory failure and multiorgan damage, underscoring the critical nature of IL-6 in disease progression [35,36,37,38]. A study on 89 COVID-19 patients with lung damage of varying degrees revealed that the levels of IL-6 signaling components (IL-6, sIL-6R, and sgp130) correlated with lung damage. Korotaeva et al. [39] suggested that the classical IL-6 signaling pathway is predominant in patients with mild or moderate pulmonary impairment, as evidenced by CT scans. In contrast, the trans-signaling pathway prevails in patients with moderate-to-severe impairment. Severe pulmonary impairment occurs when dysregulation of IL-6 regulatory mechanisms becomes evident [39]. Moreover, Drost’s study demonstrates that IL-6 and its downstream pathways are causally linked to endothelial glycocalyx (eGC) damage in inflammatory conditions, including COVID-19 and bacterial sepsis. The eGC contributes to maintaining microcirculatory homeostasis and also plays a key role in regulating the redox state, and therefore its dysfunction would lead to edema and significant organ damage. This study demonstrated that in vitro pharmacological blockade of IL-6 protects against such damage induced by sera from affected patients [40].

Elevated IL-6 levels play a pivotal role in cytokine storms associated with severe COVID-19, contributing to ARDS, multiorgan failure, and coagulation abnormalities, which manifest as increased thrombin generation and platelet activity. This pivotal cytokine induces the production of coagulation factors such as fibrinogen and factor VIII, and increases vascular permeability by enhancing VEGF secretion [41]. VEGF promotes angiogenesis and endothelial cell proliferation, exacerbating vascular issues. This proinflammatory state, marked by high IL-6 levels, is linked to clinical deterioration and higher 60-day mortality in COVID-19 patients [42,43].

In contrast, IL-6 also possesses anti-inflammatory properties that are crucial for regulating the immune response and promoting tissue repair [44]. It can induce the production of anti-inflammatory cytokines such as IL-10 and soluble IL-6 receptors, which help to mitigate excessive inflammation [45]. Additionally, IL-6 maintains immune tolerance and prevents overactive immune responses by keeping a balance in the differentiation of T cells: combining its stimulation with TGF-β enhances Th17 cell production, while stimulation with TGF-β alone supports the differentiation of regulatory T cells (Tregs) [46,47]. This is important in COVID-19 patients, because imbalance in the Treg/Th17 ratio raises the risk of respiratory failure [44,47]. These anti-inflammatory actions of IL-6 are particularly important in the resolution phase of inflammation, where they aid in tissue recovery and prevent further damage [48].

The dual role of IL-6 in COVID-19 underscores the complexity of its function within the immune system. While its proinflammatory actions are essential for an effective immune defense, the dysregulation of IL-6 signaling can lead to severe complications, including systemic inflammation and immune thrombotic processes [45] Conversely, its anti-inflammatory effects are necessary for the resolution of inflammation and repair of tissue damage. This dichotomy presents a therapeutic challenge, as interventions targeting IL-6, such as tocilizumab and sarilumab, must carefully balance harmful inflammation without suppressing beneficial immune responses [49,50].

While at the beginning of the pandemic, studies identified the serum values of the various biomarkers and aimed to evaluate their usefulness in diagnosis, prognosis, or the early initiation of biological therapy, in the third year of confronting this virus, the researchers shifted the focus to the specific genetic variations of *IL6* and how these changes could contribute to susceptibility to a severe form of the disease. Therefore, research indicated that individuals with certain genetic variations in the *IL6* gene, like the GG genotype of rs1800795, tended to have higher IL-6 levels and were more likely to experience severe COVID-19 or increased mortality [51]. Meanwhile, other gene variants, such as the C allele of rs1800795 (cytosine at the −174 position of the promoter was encoded by the C allele), have been linked to increased susceptibility to infection, but not necessarily to more severe disease [52]. Additionally, some haplotypes combining rs1800797 and rs1800795 appear to offer protection against COVID-19 by reducing the likelihood of infection or severe illness [53]. These findings suggest that *IL6* variants are important factors in COVID-19 outcomes, potentially serving as genetic markers to predict disease severity and guide treatment strategies.

#### 2.1.2. TNF-α

To protect the host from SARS-CoV-2 infection, alongside IL-6 and IL-1, TNF-α is another key cytokine involved in orchestrating proinflammatory responses and promoting immune cell infiltration [54]. This pyrogenic cytokine is produced by monocytes and CD8+ T cells, but also by dendritic cells (DCs), macrophages, Th1 and Th17 cells, epithelial cells, and endothelium cells during acute inflammation or infection [55,56]. It functions through its receptors, TNFR1 and TNFR2, triggering pathways that lead to inflammation, apoptosis, and cellular proliferation [55]. Even though in the context of viral infections, TNF-alpha is crucial for initiating and sustaining inflammatory responses that help control the infection, in COVID-19, inflammation driven by TNF-α, if excessive, can lead to significant tissue damage and progressively contribute to the development of lung fibrosis. This process eventually causes conditions such as pneumonia, pulmonary edema, and acute respiratory distress syndrome [54].

Among the various proinflammatory cytokines identified in individuals with COVID-19, TNF-α and IL-6 are present at higher levels compared to other cytokines [57]. Consequently, these cytokines were investigated as biomarkers for forecasting severe cases of COVID-19. Some studies demonstrated consistently higher levels of TNF-α than IL-6 in severe COVID-19 patients and those with underlying comorbidities such as obesity, chronic heart failure, and hypertension [58,59,60]. Others found that TNF-α and several other cytokines, including IL-2, IL-7, IL-10, chemokine CXCL10, CCL2, CCL3, and granulocyte colony-stimulating factor (GCSF), to be considerably higher in ICU patients than non-hospitalized patients [56,61]. Halim et al. studied the association between TNF-α, IL-6, vitamin D levels, and COVID-19 severity and mortality, and found: (1) insignificant differences in mean vitamin D levels between patients with severe COVID-19 and non-severe COVID-19, (2) insignificant increased risk of COVID-19 severity associated with TNF-α, (3) significant increased risk of COVID-19 mortality associated with TNF- α, and (4) IL-6 as an independent prognostic factor for COVID-19 severity and mortality [58]. In contrast, a study by Balzanelli et al. found that vitamin D levels were positively correlated with higher IL-6 levels and reduced estimated glomerular filtration rate (eGFR) in COVID-19 patients compared to healthy controls, and significant differences in the mean levels of these parameters were highlighted between the SARS-CoV-2-infected subjects and the control groups [62]. Another systematic review with meta-analysis performed by Udomsinprasert et al. found that while systemic levels of IL-6 and IL-10 were significantly elevated in severe COVID-19 patients compared to those with non-severe cases, TNF-α levels did not exhibit a similar increase [63].

Given that IL-1β, TNF-α, and IFN-γ can induce cell death in various cell types and the molecular mechanisms through which these cytokines contribute to cytokine storms remain largely unclear, Karki et al. suggested a new paradigm for defining the mechanism through PANoptosis (cytokine-mediated inflammatory cell death). Thus, TNF-α and IFN-γ-mediated PANoptosis perpetuate cytokine storms [64]. Their study showed that amongst several cytokines tested, only TNF-α and IFN-γ synergism induced inflammatory cell death or PANoptosis that is dependent on the IRF1 and NO signaling axis. By identifying this critical inflammatory cell death pathway downstream of TNF-α and IFN-γ, they revealed several potential drug targets for further investigation in the context of COVID-19 and other infectious and inflammatory diseases characterized by cytokine storms and the involvement of TNF-α and IFN-γ. Their discovery of PANoptosis and its role in disease pathology addresses a critical unmet need and provides a foundation for developing evidence-based therapeutic strategies to combat not only COVID-19 disease but also other diseases, as TNF-α and IFN-γ shock in mice mirrors cytokine storm syndromes in general [64].

From another perspective, new insights suggest that genetic polymorphisms play a role in modulating variability in host immune responses to SARS-CoV-2 and the development of CS. Research by Eldesouki et al. (2024) identified a significant correlation between elevated D-dimer levels and the −1082 GG genotype of the TNF-α gene [65]. Furthermore, the GA genotype of TNF-α-308, along with the AGG and AAA haplotypes, was found to be strongly correlated with improved survival outcomes [65].

#### 2.1.3. IL-1β

IL-1β, a proinflammatory cytokine, is involved in initiating and amplifying the immune response to infectious agents. It binds to the IL-1 receptor (IL-1R), initiating a signaling pathway that results in the production of other inflammatory mediators and the recruitment of immune cells to the site of infection or injury. Therefore, it contributes to immune cell recruitment and activation, modulation of adaptive immunity, and production of inflammasome-induced cytokines. This profoundly impacts acute and chronic autoinflammatory disorders in response to infection [46,66]. In the same time, IL-1α and IL-36, as well as other members of the IL-1 family, are correlated with skin diseases [67]. The IL-1 receptor antagonist (IL-1Ra), induced by STAT3, serves as a competitive inhibitor that binds to IL-1R, preventing IL-1β from activating proinflammatory signaling pathways. In addition, IL-6 induced by IL-1β assists in the synthesis of liver CRP in acute-phase responses [68]. Lücke et al. documented a protective role of intestinal IL-1β during SARS-CoV-2 infection [69].

IL-1β is produced by activated macrophages, monocytes, dendritic cells, and endothelium cells [46,70]. In the context of COVID-19, IL-1 is considered a critical mediator of the inflammatory response, contributing to CS and severe disease outcomes. IL-1β significantly impacts the immune response by activating T cells and natural killer (NK) cells, enhancing their cytotoxic activity. In cases of severe COVID-19, as opposed to moderate instances, there is a notable association between elevated IL-1β levels and phenomena such as hypercoagulation and disseminated intravascular coagulation [70,71].

Clinical and laboratory findings suggest that SARS-CoV-2 may promote the activation and maturation of IL-1β, which in turn can lead to the activation of other proinflammatory cytokines like IL-6 and TNF-α. Additionally, activity of IL-1β is implicated in various conditions, including Th1 cell activation, acute respiratory distress syndrome (ARDS), fever, macrophage activation syndrome, and cytokine storms. This cascade contributes to the severe inflammatory response observed in COVID-19 patients. Research has shown that peripheral blood mononuclear cells (PBMCs) from individuals with COVID-19 display inflammasome signatures linked to IL-1β, as evidenced by flow cytometry and single-cell transcriptomic analyses. These findings emphasize the critical role of IL-1β in driving the inflammatory response in COVID-19 patients [68,70].

Emerging evidence suggests that some patients experience lingering symptoms long after the acute phase of COVID-19, a condition commonly referred to as long COVID. The phenomenon, called post-acute sequelae of COVID-19 (PASC), may be more closely linked to persistent cytokine dysregulation, including sustained IL-1β activity and elevated levels of IL-6 and TNF-α, rather than the emergence of post-COVID-19 autoantibodies [72]. Future research should focus on understanding the mechanisms behind long COVID and the potential of targeted therapies to mitigate these effects. Gomes et al. found that individuals who had COVID-19, especially those with long COVID-19, had significantly higher levels of inflammatory cytokines compared to healthy people who had never been exposed to the virus. The study identified IL-6, TNF, IL-1β, IL-10, and IL-2 as the five most distinctive cytokines that could effectively differentiate between individuals who had COVID-19 (including those with long COVID) and those who had never been exposed to the virus. Among these, IL-1β as well as IL-6 emerged as particularly significant markers, especially in long COVID-19 patients, pointing towards their key role in driving persistent inflammation and ongoing symptoms following the acute phase of infection [73].

Based on the levels of IL-1β, TNF-α, and MIP-1α measured one month after the onset of the disease, Alonso-Domvínguez et al. managed to develop a highly accurate model for the early prediction of post-COVID-19 symptomatology [70,74]. Their findings suggested that a persistent inflammatory state one month after the onset is associated with elevated levels of specific cytokines (IL-1β, MIP-1α, and TNF-α), but these results need to be confirmed on larger cohorts [74].

### 2.2. Correlation Between CS and Gut Microbiota in COVID-19

The gastrointestinal tract is recognized as the most extensive immunological structure within the human body, with the gut microbiota playing a key role in regulating the host’s immune responses, protecting against toxins, and supporting nutritional metabolism [75,76]. Therefore, dysbiosis has been associated with numerous health conditions, such as inflammatory bowel disease, functional gastrointestinal disorders, viral infections, allergies, obesity, and mental health disorders [76].

Studies have demonstrated that the gut microbiota influences lung health via a significant interaction known as the “gut–lung axis” [77,78,79]. Disruptions in gut ecology can heighten susceptibility to respiratory illnesses, as demonstrated by studies linking inflammatory bowel disease (IBD) with increased risk of respiratory tract infections [76]. This gut–lung axis operates in both directions: metabolites produced by the gut microbiota can influence lung function through circulation, and lung inflammation can in turn impact the gut microbiota [75].

As seen in this review and others, the literature abounds with studies that correlate severe forms of illness or even patient death due to SARS-CoV-2 infection with host genetic factors, advanced age, male gender, comorbidities such as chronic pulmonary and cardiovascular diseases, diabetes mellitus, and obesity, as well as elevated levels of cytokines (IL-6, TNF-α, and IFN-γ) or other serum biomarkers. However, the significant variability in the clinical presentation of this disease remains incompletely explained and understood [80]. Therefore, recent research is starting to connect the gut microbiome with the underlying mechanisms of COVID-19. Evidence indicates that alterations in gut microbiota may lower ACE2 expression, which can impact viral entry and replication. Additionally, the gut barrier dysfunction observed in COVID-19 can result in heightened systemic inflammation and an increased susceptibility to respiratory infections [81]. Several studies evaluated the intestinal microbiota from COVID-19 patients, and although the number of patients was reduced and the studies had their limitations, specific changes in the microbiome of COVID-19 patients were observed. It was reported that the fecal microbiome exhibited a decline in bacterial diversity and a reduction in the population of short-chain fatty acid (SCFA)-producing bacteria, particularly from the *Lachnospiraceae*, *Ruminococcaceae*, and *Eubacteriaceae* families. Conversely, there was an increase in opportunistic pathogens from the Enterobacteriaceae family compared to the microbiome of healthy individuals. Specifically, the levels of *Faecalibacterium*, *Eubacterium*, *Coprococcus*, *Ruminococcus*, *Lachnospira*, and *Roseburia* were reduced, while the presence of Enterococcus, Rothia, and Lactobacillus was elevated. Other authors revealed significantly lower bacterial diversity and an increased presence of opportunistic pathogens, including *Streptococcus*, *Veillonella*, *Fusobacterium*, and *Escherichia*, in COVID-19 patients when comparing them with healthy controls or seasonal flu patients. Moreover, those with SARS-CoV-2 infection had higher fecal levels of the proinflammatory cytokine IL-18 compared to those with seasonal flu. Hospitalized COVID-19 patients exhibited a greater abundance of *Enterococcus faecium* and *Clostridium ramosum* compared to those hospitalized with other viral pneumonias. Thus, Zhang et al. concluded that despite the small samples, SARS-CoV-2 may still have distinct effects on the gut microbiota [81].

**Figure 1:**
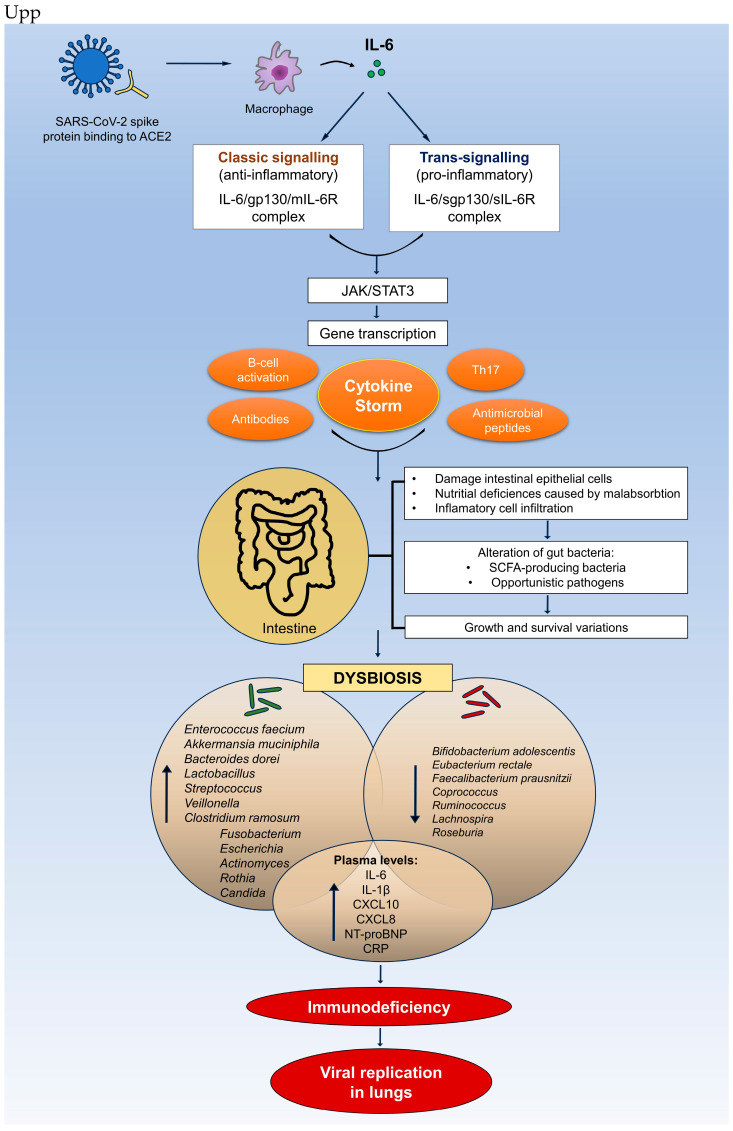
The role of IL-6 signaling in cytokine–microbiome interactions with consequences on immune response effectiveness and viral replication. For dysbiosis, the uppercase arrows indicate elevated values, while the lowercase arrow indicate a reduction in the specified bacterial species.

Positive associations with opportunistic bacterial pathogens such as *Enterococcus*, *Streptococcus*, and *Actinomyces* in male and female COVID-19 patients were also observed by Rizzello et al., along with a dominance of the Candida genus in the gut microbiota. They also found a positive correlation between unassigned Saccharomycetales fungal genera and bacterial short-chain fatty acid (SCFA) producers in COVID-19 patients, along with a negative association with the proinflammatory genus *Bilophila*. Notably, none of the patients harboring these Saccharomycetales genera required admission to the high-intensity care unit [79]. It is well known that increased severity in SARS-CoV-2 infection is due to systemic inflammation and tissue damage caused by cytokine storms more than the virus itself. Yeoh et al. suggests that the gut microbiota composition plays a crucial role in modulating the immune response in COVID-19 [82], influencing the severity of the disease through its impact on cytokine production and tissue damage. Specifically, plasma levels of inflammatory cytokines and markers of tissue damage, such as CXCL10, IL-10, TNF-α, and others, were significantly linked to the gut microbiota profile, with these markers increasing in patients with more severe disease states. Notably, certain gut bacteria that were depleted in COVID-19 patients, including *Bifidobacterium adolescentis*, *Eubacterium rectale*, and *Faecalibacterium prausnitzii*, negatively correlated with key inflammatory cytokines like CXCL10 and IL-10. These bacteria are known for their immunomodulatory roles, suggesting their depletion may contribute to the dysregulated immune response seen in severe COVID-19. Conversely, species such as *Bacteroides dorei* and *Akkermansia muciniphila*, which were enriched in the COVID-19 cohort, positively correlated with proinflammatory cytokines IL-1β, IL-6, and CXCL8, potentially exacerbating inflammation [82]. The cytokine–microbiome interactions are illustrated in Figure 1.

In another study involving 60 COVID-19 patients, elevated levels of zonulin—a protein involved in regulating the tight junctions of the gastrointestinal tract—were linked to higher mortality, more severe illness, and increased levels of systemic inflammation markers, such as IL-6. This finding underscores the connection between compromised intestinal barrier function and the severity of COVID-19 [83].

Nagata et al. conducted shotgun metagenomic sequencing and metabolomics on stool samples, along with cytokine analysis of plasma, from 112 hospitalized COVID-19 patients and 112 matched healthy controls. The study identified numerous interconnections between cytokines, metabolites, and gut microbes in COVID-19 and its complications, with fewer associations found in gastrointestinal complications. These findings highlight microbiota-driven immune responses that vary among different organ systems and underscore the role of the gut–lung axis in COVID-19 [84]. Zhang et al. [85] conducted a study to explore the functional profile of the gut microbiome in COVID-19 patients before and after recovery. Using shotgun metagenomic sequencing on fecal samples from 66 antibiotic-naïve COVID-19 patients and 70 control subjects, they found that the gut microbiome in COVID-19 patients exhibited impaired biosynthesis of short-chain fatty acids (SCFAs) and L-isoleucine. Notably, this impairment continued for more than 30 days following recovery. Targeted fecal metabolite analysis confirmed that COVID-19 patients exhibited significantly lower concentrations of SCFAs and L-isoleucine both before and after recovering form disease [85]. This impairment was significantly associated with disease severity and elevated plasma levels of CRP, CXCL10, and NT-proB-type natriuretic peptide. The study highlights that the gut microbiome’s reduced ability to produce SCFAs and L-isoleucine persists after recovery and is closely linked to the immune response in SARS-CoV-2 infection (Figure 1), emphasizing the critical role of gut microbial functions in the disease’s pathogenesis and outcome [85].

Given their role in regulating both gut and lung environments, probiotics are being investigated as adjuvant treatment against COVID-19. Clinical trials have demonstrated that *Lactobacillus acidophilus* and *Lactobacillus plantarum* can influence cytokine release, offering immunomodulatory benefits [76]. Several studies have indicated that diet, probiotics, microbiota-derived metabolites, and fecal microbiota transplantation might enhance antiviral responses and improve clinical outcomes in COVID-19, potentially by modulating the gut microbiota. However, many of these findings are based only on associative and retrospective analyses. Additional animal and clinical studies are needed to clarify the mechanistic pathways underlying the therapeutic effects of these microbiota-based interventions [81].

Phytotherapy has also been explored as a potential treatment option for COVID-19. Liu et al. investigated the effects of two food-derived immunomodulatory compounds—ajoene-enriched garlic extract (AGE) and sulforaphane (SFN), found in cruciferous vegetables—on anti-inflammatory and immune responses in a mouse model of SARS-CoV-2-induced acute lung injury. Their study demonstrated that both agents produced comparable anti-inflammatory and immunomodulatory effects to those of neutralizing monoclonal antibodies targeting the interleukin 6 receptor (IL-6R). This suggests that AGE and SFN could be promising, safe, and cost-effective candidates for COVID-19 treatment [86].

### 2.3. Current Diagnostic Methods for Identifying COVID-19 Cytokine Storm

Laboratory testing plays a crucial role in diagnosis, prognosis, and staging of COVID-19. Numerous studies have been performed on different clusters of patients stratified by disease severity or the presence of comorbidities, with conclusions drawn primarily from laboratory biomarker panels, rather than clinical evaluations. Moreover, possible multiorgan involvement in COVID-19 leads to a variety of complications, which are evaluated through laboratory assessments and used to guide clinical decision-making. Some relevant examples include: (a) neuron-specific enolase used to differentiate patients developing dyspnea [87], (b) procalcitonin, neutrophil-to-lymphocyte ratio, cluster of differentiation T cells CD25+, CD4+, CD3+, CD127−, and inflammatory markers, including cytokines and dysregulated endothelial function markers, predict disease severity [88,89], (c) cardiac markers reflect poor prognosis [90,91], and (d) high HDL-C levels are associated with a higher survival rate [92]. To further advance clinical utility and better stratify mortality risk, COVID scores have been developed based on the most prognosticative [93,94], including machine learning models with an accurate prediction of severity or outcome [95,96,97]. To identify the most effective laboratory markers and clinical parameters for screening and diagnosing COVID-19, a recent study created over 300,000 models employing various artificial intelligence algorithms, including neural networks, k-nearest neighbors, support vector machines, and extreme gradient boosting. The results showed that a cytokine profile alone has similar accuracy to a profile including other laboratory and clinical data, leading to models with excellent values for sensitivity (95.6%) and specificity (98.1%) [98]. Therefore, considering the utmost importance of immune dysregulation as a mainstay of COVID-19, the cytokine signature has been intensely studied in these patients [99,100,101,102].

All suspected cases of cytokine storm require a comprehensive assessment of infection, liver and kidney function evaluation, acute-phase protein measurement, complete blood cell count, and a cytokine profile as soon as possible [1]. A specific cytokine fingerprint for COVID is possible; however, multiple different causes of CS should always be considered. Diorio C. et al. highlighted increased levels of interferon-γ in CS due to CAR T-cell therapy, while reporting high levels of interleukin-1β and markers of endothelial damage in patients with systemic infection [103]. Kessel et al. reported a significant decrease in soluble Fas ligand levels within the context of COVID-19-associated CS. Conversely, non-COVID-19 secondary HLH exhibited activation of the IL-18–IFN-γ axis alongside elevated IL-1 receptor antagonist and IL-8 concentrations [104]. These findings underline a distinct immunological profile, which has been confirmed by other studies [105,106]. Specific cytokine cutoffs are hard to establish. In most studies, researchers compare cytokine levels in patients with COVID to baseline values from a healthy cohort [107] or cytokine levels between different stages of severity [108], maybe overlooking the positive contribution of cytokines in immune system development. A more complex study defined a range for cytokines based on post-CAR T cutoffs and afterwards tried to integrate the results in a study performed on 1484 patients with suspected or confirmed SARS-CoV-2 infection. The majority of patients presented values in the range established previously, and therefore the researchers decided to use another cutoff for each cytokine, one above the median value [57]. For example, one study group suggested a cutoff of 70 pg/mL for IL-6 for predicting severity and survival [57], while another research group proposed 80 pg/mL for predicting respiratory failure [109]. At the same time, other authors indicated that severe cases can be predicted with values for IL-6 greater than 24.3 pg/mL [110]. It is the variability in immune response characteristic to each individual, cytokines’ complex biology, and SARS-CoV-2 intricacy that pose challenges in defining specific cutoff points.

Accurate cytokine quantification is challenging because of two factors: biological relevance and cost limitations. On one side, cytokines have short half-lives (varying from minutes to hours), a fluctuating secretion pattern. and low concentrations in body fluids [111], making it difficult for their levels to remain relevant in different time frames of the pathological process. In other words, a preferred approach in patients with CS is to measure cytokine levels over time to assess their dynamics. Moreover, their stability is influenced by blood sample processing, with serum obtained with clot-activating vacutainers being the preferred option [112], together with storage at 4 °C of whole blood if centrifugation is not available right away [113]. On the other hand, the methods currently used more frequently (ELISA, PCR) usually require high-cost equipment and highly trained personnel, therefore constraining iterative cytokine trajectory tracking. Research has shifted the attention towards biosensors as a simpler and easier to use tool for detecting cytokines, as these can be used as a guide in immunotherapy for dosing and timing. Hao et al. described a dual-channel graphene–Tween 80 field-effect transistor device for measurement of IFN-γ, TNF-α, and IL-6 [114], and a more recent study introduced a highly sensitive diode-based biosensor for detecting even low levels of tumor necrosis factor α [115], suggesting its utility in the early phase of inflammation.

In a recent investigation, Calvo-Alvarez et al. focused on the cytokine response in COVID-19 patients who had not yet received vaccinations during the early stages of the pandemic. The study involved the quantification of 27 cytokines and chemokines at different time points (before, during, and at the end of the hospitalization period) using a magnetic bead-based multiplex immunoassay—Bio-Plex from Bio-Rad Laboratories, Milano, Italy. They were able to compare the levels of different molecules and identified notable increases in IL-9, RANTES (CCL5), MCP-1 (CCL2), and IP-10 (CXCL10) levels in infected individuals. The longitudinal analysis revealed a gradual reduction in IL-6 and IP-10, which correlated with disease severity. The authors also investigated Th1 and Th2 cytokine dynamics in COVID-19 patients, and found that stable ratios of Th2 cytokines (IL-4, IL-6, IL-10) to IFN-γ over time indicate a dysregulated immune response, which is critical in understanding COVID-19 pathophysiology. These results highlight the intricate interplay among cytokine activity, antibody production, and clinical outcomes, emphasizing the need for a comprehensive understanding of cytokine behavior in the context of COVID-19 to inform the development of targeted therapeutic strategies [116]. We have also shown in our previous studies, using different multiplex magnetic bead-based immunoassays suitable for the Luminex-xMAP system, that sTREM-1, HGF, and MCP-1 proved to be key predictors for disease severity and mortality, along with IL-6, in a cohort of 153 COVID-19 patients with various forms of disease severity (mild, moderate, or severe) [5]. Another panel of soluble biomarkers focusing on immune checkpoints helped us to identify the prominent roles of sTIM-1 and Gal-9 levels at hospital admission in discriminating between survivors and non-survivors. Moreover, a strong association of sTREM-1 with sCD40 characterized Delta-infected patients when compared to the Omicron cases, an association that eventually correlated with increased disease mortality [6]. Similar approaches to identifying soluble mortality predictors of COVID-19 have involved the use of classical flow-cytometry bead arrays, and extended panels investigating multiple pro-and anti-inflammatory cytokines (e.g., Il-1β, IL-2, IL-4, IL-6, MCP-1, IP-10, IL-8, IL-12, IFN-γ, TNF-α) confirmed previously reported findings [117,118].

Additional commercial cytokine chemiluminescence immunoassays used on fully automated analyzers such as the Siemens IMMULITE or Roche Diagnostics Cobas systems have been used to precisely quantify cytokine levels at hospital admission or in longitudinal studies of IL-1β, IL-2R, IL-6, IL-8, IL-10, and TNF-α, and eventually compare them to distinct hematological and coagulation laboratory analyses [119]. Most studies highlighted the association of neutrophil counts and increased NLR or PLR ratios to proinflammatory mediators (IL-6, MCP-1, and TNF-α levels) concomitant with coagulation dysregulation revealed by increased levels of D-dimers.

### 2.4. Genetic Variations Characterizing the Cytokine Storm

The impact of COVID-19 differs widely among individuals, even when they share the same working or family environment. While some individuals develop severe symptoms, others experience only mild illness or remain asymptomatic. This variability is likely due to a combination of factors, including differences in immune system responses, genetic predispositions, underlying health conditions, age, and sex. Genetic variations, such as differences in cytokine production or receptor expression, may play a crucial role in shaping the immune response, potentially leading to more severe outcomes in certain individuals. For instance, genetic variations caused by single-nucleotide polymorphisms (SNPs) might explain the different forms of COVID-19 severity. As mentioned earlier, for IL-6, two major polymorphisms are described, rs1800795 (genotype G174C) and rs1800796 (genotype C572G). The first polymorphism (G174C) was confirmed by multiple studies to be positively associated with IL-6 overexpression and subsequent increased systemic inflammation, as well as with pneumonia severity in COVID-19 patients [120,121,122]. The high expression of IL-6 is even more amplified when the expressions of *IL10* and *IL6R* are downregulated. Importantly, in the recent study of Balzanelli et al., the SNP expression of the *IL6R* gene (rs2228145) with the A/A genotype was shown to be associated with lower expression, but higher prevalence among COVID-19 patients, indicating a higher degree of infection. Similarly, IL10 rs1800896 with the genotype A/A, indicative of lower protein expression, was more frequent among COVID-19 patients than healthy controls [122]. The presence of SNPs in the promoters of *TNFA* and *IFNG* genes also modulates their abnormal expression of TNF-α and IFN-γ, characterizing cytokine storms in response to pathogenic insults and ultimately leading to multiple organ failure. Indeed, both the SNP rs1800629 of *TNFA* and the SNP 2430561 of *IFNG* were associated with increased susceptibility to pulmonary infections. A study of Balzanelli et al. also highlighted that the GG polymorphism of *TNFA* and A/A polymorphism of *IFNG* showed higher prevalence among COVID-19 patients. Since vitamin D was suggested to influence the course of COVID-19, studies also focused on identifying the variants susceptible to SARS-CoV-2 infections of the vitamin receptor gene (*VDR*). VDR expression controls and regulates both vitamin D absorption and activity via specific intracellular signaling pathways. For *VDR*, the following polymorphisms were associated with a significantly higher risk of SARS-CoV-2 infection: Fok1 polymorphism (rs2228570) with genotype T/C and Bsm1 polymorphism (rs1544410) with genotype C/C [122]. Importantly, many of the polymorphisms mentioned increased the mortality risk in SARS-CoV-2-infected individuals [62,123,124].

Thus, it is now known that SNPs in genes encoding proinflammatory cytokines, such as IL-6, TNF-α, and IFN-γ, may result in either heightened or dampened immune responses, potentially making some individuals more prone to severe inflammation and complications, while others experience milder symptoms or remain asymptomatic. These genetic variations may lead to differences in susceptibility to developing cytokine storms or other severe outcomes, helping to explain the heterogeneous clinical manifestations of COVID-19 observed even among those with similar exposure levels. Understanding these genetic factors could provide insights into individualized risk profiles and potential therapeutic strategies.

### 2.5. Advances in Cytokine Storm Therapy Management

Without argument, cytokines are one of the mainstays in the immune system. The problem is an overproduction that leads to hyperinflammation and causes detrimental systemic effects. Balance is key, and knowing the precise time to intervene with therapy has proven crucial in COVID-19.

In the early stages of COVID-19, when viral loads are high and the adaptive immune response is not yet effective, treatments targeting viral replication proved to be most effective, such as antiviral therapies based on administering remdesivir, nirmatrelvir–ritonavir, or molnupiravir and passive immunity options like anti-SARS-CoV-2 antibodies and convalescent plasma. Timely initiation of these antiviral treatments within the first 3–5 days of symptom onset is crucial in patients experiencing mild-to-moderate COVID-19 at high risk of progressing to severe illness [125,126,127,128]. In the later stages of COVID-19, particularly in patients who develop severe or critical disease, the primary cause of tissue and organ damage is thought to be an excessive and abnormal inflammatory response (often referred to as the cytokine storm), rather than direct viral replication. At this point in the disease course, targeting the underlying inflammation becomes a key therapeutic strategy, and thus anti-inflammatory treatments such as IL-6 inhibitors (e.g., tocilizumab), JAK inhibitors, or corticosteroids have proved beneficial by dampening the overactive immune response, particularly in patients requiring intensive care support (as recommended by the Infectious Diseases Society of America (ISDA) guidelines [127]). Early intervention with these agents during the critical phase could thus mitigate the progression of inflammation-driven complications, such as acute respiratory distress syndrome (ARDS) or multiorgan failure.

Initial studies on early administration of tocilizumab in moderately ill patients did not stop disease [129,130,131] and neither did late administration in severely ill patients [132]. It was only later on that a large meta-analysis (REACT) reported that tocilizumab can indeed reduce all-cause 28-days mortality rate, but only in association with corticosteroids [133]. Time has proven the importance of early administration of IL-6R antagonists to prevent CS and lower mortality rates [134], while new treatments are currently being researched, like the selective Janus kinase 1/2 inhibitor baricitinib. In 2022, the RECOVERY trial of 10,852 patients reported a significant reduction in mortality following baricitinib administration [135], proving its efficacy in managing COVID-19. The results of studies involving this molecule show similar outcomes [136] or even better ones [137] when compared to tocilizumab. These conflicting findings suggest the necessity of evaluating and validating the results on greater cohorts. A recent systematic review and meta-analysis that included 10 studies reported a significantly lower incidence of adverse events in patients treated with baricitinib over patients treated with tocilizumab. However, the same study showed no change in mortality rates or hospital length of stay when compared with patients receiving the classical treatment of tocilizumab [138]. To investigate the potential efficacy of combining the two molecules, researchers found no significant benefits beyond a reduced rate of ICU admissions in the combined-therapy group [139]. Going a step even further and adding an inhaled DNase that dissolves thrombogenic neutrophil extracellular traps, a research group reported shorter hospitalization time and in-hospital mortality, as well as improved survival rate in a small cohort of COVID-19 patients [139], suggesting that approaching immune thrombosis pathways could represent a valuable lead to follow in further studies. Given the superior efficacy of combined tocilizumab and corticosteroid therapy, researchers sought to determine whether baricitinib could replicate these benefits or generate better outcomes when used in conjunction with corticosteroids. A complex and recent meta-analysis on 13,549 patients from 27 randomized trials reported outcomes similar to the administration of tocilizumab and corticosteroids [139].

In a study conducted by Egidia Miftode et al., a critical factor in achieving the therapeutic benefits of tocilizumab in COVID-19 patients, both with and without diabetes mellitus, was the timing of administration relative to the onset of the cytokine storm. Delayed initiation of therapy, particularly after substantial respiratory impairment, significantly diminished survival chances. The study reported significantly elevated IL-6 levels in both diabetic and non-diabetic patients with severe COVID-19, reflecting the pathological effects of the virus. Clinical and biological improvements were observed following tocilizumab treatment, especially when administered early, suggesting that prompt initiation of immunomodulatory therapy at the first signs of cytokine storm is crucial. The risk of adverse outcomes was directly related to the degree of delay in treatment initiation, with 23.3% of diabetic patients succumbing to the disease despite the administration of IL-6 antagonist therapy. Moreover, tocilizumab not only reduced inflammation but also provided an important benefit in preventing the development of refractory pneumonia in patients with COVID-19-associated pneumonia [140].

During the early stages of the COVID-19 pandemic, concerns arose about the use of non-steroidal anti-inflammatory drugs (NSAIDs) like ibuprofen, due to fears they might exacerbate the disease by increasing the expression of ACE2 receptors, which serve as the entry point for the virus into human cells [139,141]. These concerns were significantly influenced by correspondence published in *Lancet Respiratory Medicine*, which suggested that angiotensin-converting enzyme inhibitors (ACEIs), angiotensin-receptor blockers (ARBs), and ibuprofen could increase the risk of SARS-CoV-2 infection and complications. As a result, at that moment, some health authorities recommended avoiding NSAIDs, favoring alternatives such as paracetamol for managing symptoms. However, subsequent studies and reviews [142,143,144], including assessments by the World Health Organization, found no substantial evidence linking NSAIDs to worsening COVID-19 outcomes or increasing susceptibility to infection. Current guidelines now indicate that NSAIDs can be safely used in COVID-19 patients to manage their health conditions and symptoms [145]. Moreover, NSAIDs reduce the cytokine release during viral stimulation, and their administration is associated with lower mortality rates in animal models [146]. These molecules modulate CS in COVID-19, notably meloxicam, which exhibited a downregulatory effect on cytokines (IL-6, CCL2, GM-CSF, CXCL10, IL-2, and TNF-α) [147].

Dexamethasone has also transitioned from a cautious consideration to a cornerstone treatment for severe COVID-19. The turning point came with the results of the RECOVERY trial, published in June 2020. This large randomized controlled trial demonstrated that dexamethasone significantly reduced mortality in patients with severe COVID-19 who required respiratory support, including those on mechanical ventilation and those receiving supplemental oxygen. The trial showed a 35% reduction in death for patients on ventilators and a 20% reduction for those needing oxygen. These findings marked a pivotal moment in the treatment of COVID-19, leading to widespread adoption of dexamethasone as a standard of care for severely ill patients [148]. Additionally, the modulation of interferon-related signaling was beneficial in these patients with increased severity, but at the same time, inhibiting interferon responses is detrimental to patients in the early phase, when strong interferon activity is essential to the favorable progression of COVID-19 [148].

Importantly, other research groups are currently focusing on investigating whether selectively blocking IL-6 trans-signaling could reduce symptoms and severity in COVID-19 while preserving the host defense activity of classical IL-6. The first selective inhibitor of IL-6 trans-signaling targets the sgp130 unit and has already shown promise as a therapeutic target across various preclinical disease models. Ettich et al. proposes a hybrid soluble gp130/spike–nanobody fusion protein that inhibits not only IL-6 trans-signaling but also cellular infection with SARS-CoV-2 [148]. A recombinant version of this protein, named olamkicept, showed positive outcomes in phase II clinical trials for inflammatory bowel disease [35], and it remains to be seen if this molecule can be used in COVID-19. A recent animal study confirmed these findings and demonstrated that the same protein, sgp-130Fc, may reduce the proinflammatory effects of IL-6 [149].

## 3. Conclusions and Open Questions

Even though much has been written about the cytokine storm in COVID-19 and there is a general understanding of the cytokines involved, the exact mechanisms through which this hyperactive response is triggered, as well as how the cytokines interact with each other and with other components of the immune system, remain incompletely elucidated. Furthermore, there is still no consensus on the most effective set of biomarkers for clinicians to detect the early onset of this cytokine storm and intervene in a timely and appropriately manner. Although the literature indicates that CRP and IL-6 are the most studied biomarkers, with their efficacy demonstrated in various studies and clinical trials, it remains unclear what the most effective approach is to utilize these markers for personalized therapeutic management. An open question also remains as to which other markers, when combined with current indicators, might lead to the most efficient case management strategies. Additionally, the involvement of demographic, genetic, environmental factors, the microbiome, and patient comorbidities in the triggering and/or maintenance of this abnormal immune response induced by the presence of the SARS-CoV-2 virus in the body is still underexplored.

Although conceptually defined—both in a noninfectious and nonspecific infectious context—some time ago, the cytokine storm prompted multiple debates only with the onset of the COVID-19 pandemic. Thus, clinicians urgently needed clarifications regarding the conditions under which the inflammatory cascade induced by the SARS-CoV-2 virus is generated, as well as the methods to identify such a complication and provide aid to the patient. This acute need led to an extraordinary mobilization of the medical scientific community, producing abundant literature with small, medium-sized, large, or even clinical trials, the results of which sometimes diverged from our real-time observations as clinicians at the patient’s bedside. The clinical and paraclinical diagnostic criteria, which were modified in real time with the new discoveries about this virus and the disease’s pathophysiology, underpinned the early initiation of therapy with interleukin antagonists (tocilizumab and anakinra) in carefully selected patients. The study of inflammatory phenomena during SARS-CoV-2 infection led to the establishment of algorithms, through the application of which the vital prognosis could be significantly improved. Looking ahead, we can assert that the pathophysiological model, the understanding of the relationships between proinflammatory and anti-inflammatory molecules, as well as therapies to prevent/counteract these imbalances, extensively researched in the literature during this pandemic and post-pandemic period, can form the foundations for research into the approach of other infectious pathologies such as sepsis, meningitis, and endocarditis in all their complexity.

## Figures and Tables

**Table 1:** Diseases associated with cytokine storm syndrome/cytokine release syndrome.

Disease	Cause	References
Infection-induced	Viral infections: COVID-19 and MIS-C, HIV (AIDS infection and secondary infections/malignancies), EBV, HHV6, CMV, hemorrhagic fever viruses, influenza viruses	[7,8,9]
	Bacterial infections: *Mycobacterium tuberculosis*, *Rickettsia* spp., *Ehrlichia* spp. and any other bacteria causing hematogenous infection (bacterial sepsis)	[1,7,10,11]
	Fungal (e.g., histoplasmosis) and parasitic infections (e.g., Leishmania, Plasmodium, Toxoplasma)	[7,12]
Genetic disorders	Familial hemophagocytic lymphohistiocytosis (HLH)	[1,7,13]
	Immunodeficiencies (e.g., PIK3CD, ITK)	
	Autoinflammatory/inflammasomopathies (e.g., NLRC4, CDC42)	[1,7]
Autoimmune-related	Systemic lupus erythematosus	[7,14]
	Still’s disease	[7,15]
	Kawasaki disease	[7,16]
	Idiopathic multicentric Castleman’s disease (MCD)	[1,17]
Malignancy-associated	Hematologic (e.g., leukemia, lymphoma)	[7,18]
	Solid tumors	[7]
Iatrogenic-induced	CAR-T cell therapy	[7,19,20]
	Blinatumomab therapy	[7,21]
Others	Graft-versus-host disease (first used by James L. Ferrara in 1993)	[22]
	Anaphylaxis	[22,23]
	Pregnancy	[7,24]
	Cardiac bypass/ECMO circuit	[7,25]
	Castleman disease	[7,26]

Abbreviations: AIDS, acquired immunodeficiency syndrome; CAR-T, chimeric antigen receptor T cell; CMV, cytomegalovirus; COVID-19, coronavirus disease 2019; EBV, Epstein–Barr virus; ECMO, extracorporeal membrane oxygenation; HHV, human herpesvirus; HIV, human immunodeficiency virus; HLH, hemophagocytic lymphohistiocytosis; HSV; MIS-C, multisystem inflammatory syndrome in children.

## Data Availability

No available data.
